# Sternochondral allograft for chest wall reconstruction

**DOI:** 10.1186/1749-8090-8-S1-O236

**Published:** 2013-09-11

**Authors:** HR Davari, MB Rahim, SAA Alavi, P Mardani, R Ershadi, AA Tavakoli, S Dehghani

**Affiliations:** 1 General Thoracic Ward, Valiasr Hospital, Imam Khomeini Hospital, Tehran University of Medical Sciences, Tehran, Iran; 2 Iranian Tissue Bank Research and Preparation Center, Imam Khomeini Hospital, Tehran University of Medical Sciences, Tehran, Iran; 3 Sina Harvesting Center, Sina Hospital, Tehran University of Medical Sciences, Tehran, Iran

## Background

Chest wall reconstruction after tumor resection or infection provides diagnostic and therapeutic challenges to the thoracic surgeon. Wide resection and reconstruction is the main issue regarding oncologic aspect.

## Methods

Since the beginning of 2012 four cases who have been presented with sternal tumors and one with post cardiac surgery infection were selected for chest wall reconstruction with bone allograft. One patient had missed diagnosis as a case of breast tumor with final diagnosis of synovial cell sarcoma. The other one had upper sternum bulging. True- cut biopsy was suggestive of myxoid tumor. Third patient had bulging mass in right upper ribs adjacent to sternum with final diagnosis of fibrous tumor. Fourth patient with morbid obesity had mediastinitis post CABG surgery. She had a total sternectomy with unstable chest wall. After fully evaluation and getting consent for bone allograft they were put on the list of sternochondral bone allograft from a heart beating donor. All operations were done within 1 month with consideration body size match. Sternum was used after processing by serial culture, freezing and sterilized with Ethylene Oxide. Allograft was fixed with Titanium microfixation after tailoring to fit perfectly the chest wall defect. Local muscle flap were used to cover the grafts.

## Results

The operations were uneventful. First patient had BMI=40. She developed infection in skin and breast tissue. Her wound was managed with water jet hydrotherapy technique and negative pressure wound therapy but the allograft was left intact.

## Conclusions

This technique is a new era in chest wall reconstruction which provides good functional and cosmetic results. It is more resistant to infection than other prosthesis. Allograft procurement, body size match, and limitation in donation are major issues. It needs further studies and more cases to understand the biology of such volume bone allograft and long term result.

